# Prevalence of anti-leptospiral IgM and detection of pathogenic *Leptospira* species DNA in neonates presenting with clinical sepsis in Southwestern Uganda

**DOI:** 10.1186/s40001-022-00902-w

**Published:** 2022-12-02

**Authors:** Derick Hope, Stephen Businge, Stella Kyoyagala, Joel Bazira

**Affiliations:** 1 Present Address: Department of Medical Laboratory Sciences, Faculty of Health Sciences, Muni University, Arua, Uganda; 2 Department of Medical Microbiology, Faculty of Medicine, Mbarara University of Science and Technology, Mbarara, Uganda; 3 Holy Innocent Children’s Hospital, Mbarara, Uganda; 4 Neonatal Unit, Department of Pediatrics, Mbarara Regional Referral Hospital, Mbarara, Uganda

**Keywords:** Leptospirosis, Neonatal sepsis, IgM ELISA

## Abstract

**Background:**

Leptospirosis is an emerging neglected zoonotic disease that presents with nonspecific signs/symptoms and it can be mistaken for other diseases. Owing to limited diagnostic capacity and unawareness, the data on human leptospirosis particularly in neonates are scarce in many sub-Saharan countries. It has been underreported hindering preventive and control measures in place. The study aimed at determining prevalence of leptospirosis as a cause of febrile illness in neonates using IgM ELISA and a quantitative real-time PCR (qPCR).

**Methods:**

This was a descriptive cross-sectional study that included 103 neonatal sepsis cases whose parents/legal guardians gave informed consent. The data on demographic and clinical characteristics were collected using structured data collection form. EDTA whole blood sample was collected from the neonates by trained study nurses. From the samples, IgM ELISA was done using automated analyzers, DNA extracted and qPCR was performed using primers for LipL32, specific for the pathogenic leptospires.

**Results:**

The prevalence of anti-leptospiral IgM among the neonates as determined by ELISA was 4.3%, where all of them presented with lethargy and poor feeding. No pathogenic *Leptospira* species DNA was amplified by qPCR.

**Conclusions:**

Evidence of leptospirosis was demonstrated in neonatal sepsis cases in this study. The findings suggest considerations of leptospirosis in the differential diagnosis of neonates with sepsis. More data are needed on the real epidemiology, clinical features, and burden of leptospirosis in neonates. There is need to include intermediate pathogenic species of *Leptospira* in the diagnostic qPCR assays.

**Supplementary Information:**

The online version contains supplementary material available at 10.1186/s40001-022-00902-w.

## Introduction

Leptospirosis is a significant emerging zoonotic infection of public health concern that has been underreported particularly in developing countries and it is recognized as a neglected tropical disease affecting vulnerable populations [1–3]. The clinical presentation mimics that of other febrile illnesses often leading to misdiagnosis clinically [4, 5]. As such world health organization recommends confirmation of the disease by laboratory diagnosis in conjunction with clinical findings and exposure status [6]. For effective treatment, antibiotic therapy has to be initiated early thus necessitating early diagnosis of the infection. Leptospirosis is caused by the spirochaete of the genus *Leptospira* and the pathogen is divided into the pathogenic (P1) and intermediary pathogenic (P2) group [7–9]. Infections have been reported to be caused mostly by the P1 group, explaining why the diagnostic qPCR assays widely used detect only the P1 group. However, there are increasing reports of detection of P2 in clinical samples including in the current study site [10–12]. The reservoirs for the leptospires include rodents particularly rats, domestic animals mainly pigs but also dogs and cattle [5], all of which are common in the current study region. Seroprevalence of leptospirosis in cattle and African buffalo in the study region, Southwestern Uganda was shown to be 29.35% and 42.39%, respectively [13]. Humans get infected by direct or indirect contact with urine of infected animals where the spirochetes penetrate the humans through breaks in the skin, mucous membrane or intact conjunctival mucosa [4, 5, 14]. Another route of infection noted is through congenital transmission [6, 12, 15]. Leptospirosis is more common in risk groups that are exposed to animal reservoirs or contaminated environment, such as abattoir workers, farmers (rice and sugar cane workers), ranchers, veterinarians, sewage workers, and among individuals partaking in water sports and recreation [4, 5].

Leptospirosis has a worldwide distribution with epidemic potential. It is endemic in humid tropical and subtropical regions of the developing world [3, 6, 16]. It is regarded as disease of the poor where resource-poor regions are associated with highest burden of disease [1, 3]. The precise number of human leptospirosis cases worldwide is unknown. A systematic review in 2015 revealed that there are 1.03 million cases and 58,900 deaths each year with 2.9 million disability adjusted life years lost per annum [1, 17], placing it as a leading zoonotic cause of morbidity and mortality. The prevalence of leptospirosis in Africa in patients with nonspecific febrile illness from a systematic review of studies that did serodiagnosis ranged from 2.3 to 19.8% [18]. Owing to limited diagnostic capacity and unawareness, the data on human leptospirosis as a cause of febrile illness are scarce in many Sub-Saharan countries [16], hindering prevention and control measures. The results from a recent study in Uganda reported seroprevalence of 35% in rural western part of the country, but the study only included adults [19]. Studies conducted in Mbarara regional referral hospital (MRRH) and elsewhere in Uganda on neonatal sepsis (NS) reveal failure to isolate microorganisms in more than half of the NS cases [20, 21]. Leptospires are not routinely diagnosed or cultivated in MRRH microbiology laboratory. A study involving sequencing of 16 s rRNA (an expensive technique) in Mbarara regional referral hospital (MRRH) showed that 40% of blood samples collected from neonates with suspected sepsis had *Leptospira* [12].

The current study sought to determine the prevalence of anti-leptospiral antibodies as an evidence of leptospirosis as a cause of febrile illness in neonates presenting with clinical sepsis using IgM ELISA and detection of pathogenic *Leptospira* species DNA by quantitative real-time PCR (qPCR). Merits of IgM ELISA over other serodiagnostics include the detection of antibody in early phase of the disease, the use of single genus-specific antigen, no need to maintain live leptospires through culture, less cumbersome, and being able to be standardized [6].

## Materials and methods

### Study design and setting

This was a descriptive cross-sectional study conducted in pediatrics ward of Mbarara Regional Referral Hospital (MRRH) and Holy Innocent Children’s Hospital (HICH), Mbarara in southwestern Uganda. MRRH receives referrals from health facilities in the neighboring districts in the region and also receives patients from neighboring countries like Tanzania, Democratic republic of Congo and Rwanda. HICH is exclusively a children’s hospital, the only one in the region. The population in the study site visits MRRH/HICH whenever they experience an illness or when referred from peripheral health units. The majority of the people in the study area live in rural areas and they are subsistence crop farmers and practice livestock farming.

### Study population

The study included neonates who; presented with suspected clinical sepsis at the study sites, were from exposed contaminated environment and whose parent or legal guardian consent to participate in the study. In the current study, a neonate was defined as a newborn less than 1 month (30 days) old. Neonates who presented with fever/hypothermia and at least any one of the following; lethargy, poor feeding, full fontanel, vomiting, seizures, diarrhea, respiratory dysfunction, and jaundice were enrolled in the study. Exposure status was defined by residence near a stream of water and or its use as source of water, presence of livestock or rodents at home and parents/guardian or caretaker’s occupation that predisposes to contact with contaminated water and or animals. Participants with known underlying etiology, such as malaria or any other cause of febrile illness at inclusion or done at bedside as determined by the clinician were excluded because of the possibility of cross reaction in the IgM ELISA analysis.

### Sampling and data collection

A total of 103 participants were recruited through convenient sampling method from August, 2018 to November, 2018. An experienced and trained research nurse from each site with the guidance of a clinician noted the clinical presentation and exposure history of neonates admitted at MRRH/HICH and sought consent from parents/caretakers of neonates who fell in the inclusion criteria. Clinical and demographic data were recorded using the predesigned questionnaire from those who consented to participate in the study. The data collected included, but not limited to; sex and date of birth (age of the baby, days), duration of fever, source of water for domestic use, presence of animals at home, and signs and symptoms of the infection. From the neonates whose parents/caretakers gave informed consent, about 0.5 mL of blood was drawn aseptically by venipuncture from the arm into EDTA microtube. The blood sample was later used to obtain plasma. Plasma was chosen because of the slightly higher sensitivity for PCR assays compared with whole blood and serum [22] and would later be used for IgM ELISA.

### Laboratory procedures

The samples were analyzed at Epicentre research laboratory in Mbarara University of science and technology (MUST). The EDTA whole blood was centrifuged at 3000 rpm for 5 min to obtain plasma. 10 µl of the plasma was used for IgM ELISA to detect anti-leptospiral antibody while about 200 µl of plasma was used for DNA extraction and subsequent detection. ELISA was done using the automated set of analyzer Washer 470 and Reader 270 (BioMérieux) and the Leptospira IgM ELISA kit, EIA-4715 (DRG International, INC). Nine (9) plasma samples were excluded from ELISA analysis either because of anticipated interference due to sample properties (cloudy lipemic samples) or too little volume to proceed with DNA extraction. The procedure was performed and interpreted according to manufacturer’s instructions. For IgM ELISA, positive and negative controls were included for quality control (QC) and the test was considered valid when the QC passed. All the samples were run in duplicates, and checked whether the replicates gave the same cutoff result. Besides the reader, the wells were checked visually with reference to positive and negative control against a white background and the intensity of color formed graded according to the kit insert.

DNA was extracted from the plasma using the QIAamp^®^DNA Mini, 250 (Qiagen, German), following manufactures protocol. Internal positive and negative control samples were included in each batch of DNA extraction procedure and PCR. A real-time PCR assay using the probe-specific ‘BactoReal *Leptospira* spp, LipL32’ kit (Ingenetix, Austria) was run for the detection of the LipL32 gene found in pathogenic leptospires with the Rotor-Gene Q real-time PCR instrument according to kit instructions. The sensitivity of the initial PCR protocol was 10 target copies/PCR reactions and Ct of 24, 3. A repeat PCR was done with in-house optimized reaction. The mastermix 5× HOT FIREPol EvaGreen qPCR Supermix (Solis Biodyne, Estonia), Positive DNA controls from Institut Pasteur, and Primers; LipL32F, 5′-AAGCATTACCGCTTGTGGTG-3′ and LipL32R, 5′-GAACTCCCATTTCAGCGATT -3′ (Inqaba Biotec, South Africa) with sensitivity of 1 target copies/PCR reaction were used in the optimization of the quantitative PCR. The primers used were designed and described by Picardeau (Institut Pasteur) and colleagues [22]. The final reaction considered had 4 µl of mastermix, 0.4 µl each of forward and reverse primers (10 pmol/µl), 10.2 µl molecular grade water and 5 µl of template. The temperature profile consisted of initial denaturation at 95 °C for 12 min and 40 cycles of; 95 °C for 15 s, 65 °C for 30 s and 72 °C for 30 s (acquisition at Green channel). The analysis was done using quantitative, endpoint, and melt curve analysis at the Green channel.

### Data management and analysis

The data were entered using Epidata and imported to and analyzed using STATA version 12. It was presented by the use of pie charts and tables. Continuous variables were presented as mean ± standard deviation. Prevalence was determined as the proportion of positive samples.

## Results

### Demographic, clinical characteristics, and exposure risk

A total of 103 neonates were included in the study based on the clinical presentation and risk of exposure, of which 67 (65%) were from HICH and 36 (35%) were from MRRH. Out of the total, 53 (51.5%) were females (Table 1). The age ranged from 1 to 29 days with mean of 6.5 ± 7.4. The weight ranged from 1.7 to 6 kg with mean of 3.2 ± 0.66. The duration of illness from onset to review as reported by the mothers ranged from 4 to 336 h (14 days) and the mean was 52.1 ± 59.8. The most common sign and symptoms included fever, lethargy, poor feeding, respiratory dysfunction, seizures, and jaundice (Table 1). Also rash, vomiting hypothermia, bleeding among others were observed. Information on the mothers of the neonates indicated that 51 (49.5%) reported to have experienced fever during pregnancy. On exposure risk, 92 (89.3%) and 77 (74.8%) had rats and livestock, respectively at home with goats (56.3%) followed by cattle (31.1%) forming majority of livestock. Tap water was the main source of water, as well as springs, wells, streams, tanks, and swamps among others (Table 1).

**Table 1:** Demographic/clinical characteristics of neonates and mothers, exposure risk, and water sources

Variables			
Variable name	Category	Frequency^b^ (%)	Mean (SD)
Neonates			
Sex	Female	53 (51.5)	
	Male	50 (48.5)	
Age (days)	1–10	82 (79.6)	6.5 (7.37)
	11–20	9 (8.7)	
	21–30	11 (10.7)	
Weight (kg)	1.6–2.5	11 (10.7)	3.2 (0.66)
	2.6–3.5	70 (68)	
	3.6–4.5	19 (18.4)	
	4.6–5.5	2 (1.94)	
	5.6–6.5	1 (1)	
Clinical presentation^b^	Fever	87 (85.3)	
	Lethargy	61 (59.8)	
	Poor feeding	92 (90.2)	
	Seizures	21 (20.6)	
	Respiratory dysfunction	53 (52)	
	Jaundice	17 (16.7)	
Time from onset of illness (fever) to sample collection (hours)^a^	4–48	77 (74.8)	52.1 (59.8)
	49–96	10 (9.7)	
	97–144	1 (1)	
	> 144	9 (8.7)	
Visible bruises	Yes	58 (56.3)	
	No	43 (41.7)	
Mothers			
Fever at pregnancy	Yes	51 (49.5)	
	No	37 (35.9)	
Exposure risk and water sources			
Rats	Yes	92 (89.3)	
	No	11 (10.7)	
Livestock	Yes	77 (74.8)	
	No	26 (25.2)	
Water source	Tape water	57 (55.3)	
	Well	17 (16.5)	
	Streams/river	4 (3.9)	
	Springs	5 (4.9)	
	Others	20 (19.4)	

^a^Duration of illness at sample collection as reported by mothers/caretakers

^b^Information on clinical presentation of one participant was missing. Some variables had a few missing values as well either because mothers/caretakers had no response or such data were missing on the medical form

### ELISA results

The results of IgM ELISA showed that 4 out of 94 tested samples were reactive, all of which were from HICH. All replicates had the same cutoff result when compared with first run. And according to the set cutoff definition, clinical picture, and exposure risk, these were considered positive giving anti-leptospiral antibody prevalence of 4.3% by IgM ELISA. The samples which tested reactive for anti-leptospiral IgM on ELISA reader were all graded as + (plus one) on visual interpretation. There were four other samples with very little color development according to visual observation, but they were considered nonreactive not only based on the visual interpretation but also because they were below the set cutoff definition on the reader. These positive cases had rats and/or livestock and the duration of illness from onset (fever) to review as reported by the mothers/caretakers was 48 h for two cases, 24 h and 96 h for the other two. The age for two of the positive cases was 7 days while the others were 2 and 3 days old. Sex was equally distributed in the positive cases. All the positive cases had the clinical presentations lethargy and poor feeding. Two (2) out of 4 had respiratory dysfunction and one (1) had pustular rash.

### PCR results and analysis

**Fig. 1:**
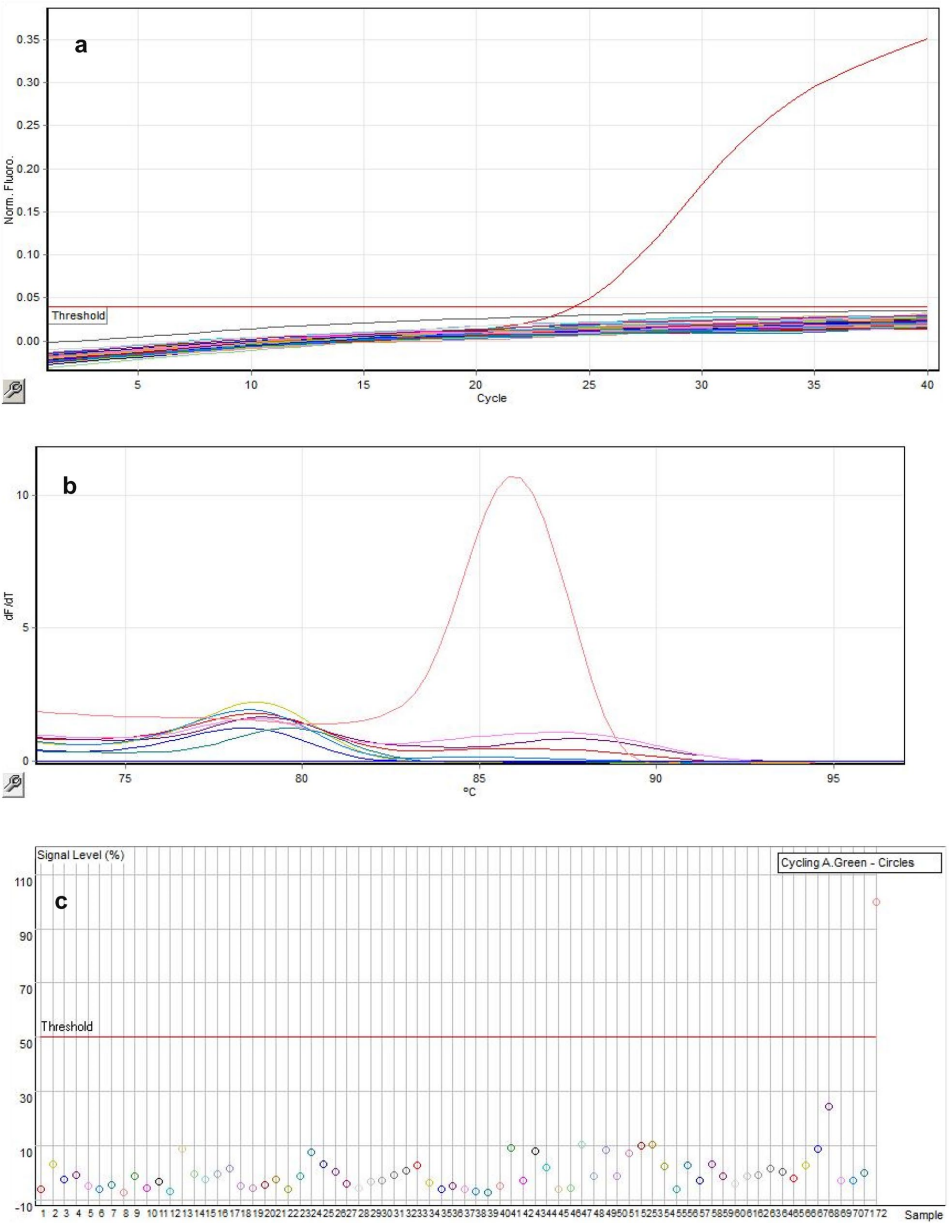
**a** Quantitative analysis, **b** Melt curve analysis, **c** Endpoint analysis

Real-time PCR was run on all the samples (103) to detect the pathogenic LipL32 gene. Out of all the analyzed samples, no pathogenic *Leptospira* was detected. Analysis was done using quantitative, melt curve, and endpoint analysis on the green channel altogether to rule out any nonspecific amplification. Using the above analyses collectively it can be seen that PCR was negative for the samples (Fig. 1).

## Discussion

This study aimed at determining prevalence of anti-leptospiral antibodies as evidence of leptospirosis using IgM ELISA and detection of pathogenic *Leptospira* DNA in neonates presenting with clinical sepsis in Southwestern Uganda. The positive cases as indicated by IgM ELISA had rats and/or livestock. These animals have been known as natural maintenance host for leptospires, shedding the spirochetes in their urine and hence transmitting to humans [5]. Further still, leptospirosis has been demonstrated to be present in animals in southwestern Uganda [13]. In neighboring Tanzania, extensive contact with cattle has been associated with higher rates of seroprevalence [23]. Contact with animals increases risk of exposure and infection. Patients may get infected through direct or indirect contact with domestic animals where transmission may occur through breaks in the skin or intact conjunctival mucosa [5]. The study showed a prevalence of 4.3% with IgM ELISA. The data on prevalence of leptospirosis in neonates are lacking in sub-saharan Africa. Serological studies have been conducted in infants and children with febrile illness in the neighboring Tanzania with prevalence of 7.7% [24] and 6.2% (probable and confirmed leptospirosis) in infants and children alone [25]. Anti-leptospiral IgM antibodies are produced first during infection and may remain for months or years [6]. However for newborns who are only less than 1 month old, detection of IgM may be suggestive of recent or current infection. The neonates with reactive IgM ELISA ranged from 2 to 7 days old in age and the duration of fever on medical review as reported by the mothers ranged from 1 to 4 days. This might suggest possible congenital transmission since the neonates had not been exposed considerably to the environment. Congenital vertical transmission is more likely to occur in the third trimester a stage when the IgG level transmitted to the fetus from the mother just begins to increase and at the same time when fetal plasma cells are fully developed [26]. Antigen-specific antibody response can be mounted in both prenatal and neonatal life but at a lower intensity. The antibody response is better to proteins than polysaccharides and it is noteworthy that antibody production in leptospirosis is mainly directed against the lipopolysaccharide [14, 26]. There has been evidence that leptospirosis can be vertically transmitted though rarely [6, 12, 15]. Detection of IgM alone is not a diagnostic confirmation, but rather should be in conjunction and consideration with clinical findings, exposure history and other laboratory findings [6]. Neonates in the study were all from environment with increased risk of exposure. A nonreactive IgM ELISA may not necessary mean absence of leptospirosis but it could be due to poor immune response as noted in neonates [26] or in the early phase of the disease when antibody levels have not been attained to a detectable amounts, which normally occurs 4–7 days after onset of the disease [5, 6]. Since antibody levels may not be detected during early disease, repeat 2–3 weeks later when antibody levels are significant is indicated for laboratory diagnosis. This will imply that there is need for a second convalescent sample, a point when patients will have been discharged with empiric therapy hence not practical for patient management in the current practice. Serodiagnosis of leptospirosis using IgM ELISA in a systematic review was found to be sensitive for use as initial screen for leptospiral infections especially in endemic areas with sensitivity and specificity of 84% and 91%, respectively [27].

In the present study, leptospires were not detected on qPCR performed. This is in contrast to the 40% prevalence in a previous study on neonatal sepsis at MRRH [12]. This could be attributed to the fact that the previous study used 16 s rRNA gene sequencing which detects the intermediary pathogenic species (P2) in addition to pathogenic species (P1). In this current study, qPCR was done targeting LipL32 gene. Studies that conducted qPCR assays to amplify the LipL32 revealed failure to detect the intermediary pathogenic strains (P2) [22, 28]. The widely utilized diagnostic qPCR assays and accessible PCR kits that target leptospiral lipL32 gene use primers specific for P1 group [22, 28, 29] hence detecting only the pathogenic strains which until recently are known to be responsible for much of the leptospirosis. There has, however, been increasing reports of the intermediate species been detected in clinical samples, including in the current study site [10–12]. These previous studies used 16 s rRNA sequencing. It is noteworthy that the prevalence of *Leptospira* spp. DNA in febrile humans in Ecuador showed that the percentage of intermediate cluster strains was higher than that of pathogenic cluster strains, that is 96% and 4%, respectively [11], while a study in Uganda showed that of the 32 detected *Leptospira* spp, 31 were intermediary species [12]. These studies reveal the high prevalence of the intermediary pathogenic species in clinical samples that could not be detected with the current qPCR assays. Other possible reasons for negative qPCR results may include neutralization of leptospires by antibodies (IgG from the mother), elimination of leptospires from blood by administered antibiotics where 13.7% of the patients in the current study received antibiotics at the time of sampling (see Additional file 1: Table S1) and possibility of PCR inhibition.

## Conclusions

The study demonstrated evidence of leptospirosis in neonates with clinical sepsis as revealed by the detection of anti-leptospiral IgM with a prevalence of 4.3%. Pathogenic species of *Leptospira* DNA was not detected in any of the samples by qPCR. The findings suggest considerations of leptospirosis in the differential diagnosis of neonates with febrile illness in endemic areas. More data are needed to determine the real epidemiology and burden of leptospirosis in neonates and understand the clinical presentations, so as to draw preventive and control measures like screening of mothers in endemic area. The results from the study points towards a need to include detection of intermediary pathogenic species of Leptospira in the diagnostic qPCR assays.

## Supplementary Information

**Additional file 1: Table S1.** Leptospirosis raw data 1-Data showing participants’ characteristics and demographic information.

## Data Availability

The datasets used and/or analysed during the current study are available from the corresponding author on reasonable request.

## Abbreviations

HICH: Holy Innocent Childrens’ Hospital
MRRH: Mbarara regional referral hospital
MUST: Mbarara University of Science and Technology

## References

[1] Costa F, Hagan JE, Calcagno J, Kane M, Torgerson P, Martinez-Silveira MS, Stein C, Abela-Ridder B, Ko AI (2015). Global morbidity and mortality of leptospirosis: a systematic review. PLoS Negl Trop Dis.

[2] Karpagam KB, Ganesh B (2020). Leptospirosis: a neglected tropical zoonotic infection of public health importance—an updated review. Eur J Clin Microbiol Infect Dis.

[3] WHO (2011). Report of the second meeting of the leptospirosis burden epidemiology reference group.

[4] Forbes BA, Sahm DF, Weissfeld AS (2007). Bailey and Scott’s diagnostic microbiology.

[5] Haake DA, Levett PN, Adler B (2015). Leptospirosis in humans. Leptospira and leptospirosis.

[6] Organization WH (2003). Human leptospirosis: guidance for diagnosis, surveillance and control.

[7] Morey RE, Galloway RL, Bragg SL, Steigerwalt AG, Mayer LW, Levett PN (2006). Species-specific identification of Leptospiraceae by 16S rRNA gene sequencing. J Clin Microbiol.

[8] Vincent AT, Schiettekatte O, Goarant C, Neela VK, Bernet E, Thibeaux R, Ismail N, Mohd Khalid MKN, Amran F, Masuzawa T (2019). Revisiting the taxonomy and evolution of pathogenicity of the genus Leptospira through the prism of genomics. PLoS Negl Trop Dis.

[9] Cerqueira GM, Picardeau M (2009). A century of Leptospira strain typing. Infect Genet Evol.

[10] Balamurugan V, Gangadhar NL, Mohandoss N, Thirumalesh SRA, Dhar M, Shome R, Krishnamoorthy P, Prabhudas K, Rahman H (2013). Characterization of leptospira isolates from animals and humans: phylogenetic analysis identifies the prevalence of intermediate species in India. Springerplus.

[11] Chiriboga J, Barragan V, Arroyo G, Sosa A, Birdsell DN, España K, Mora A, Espín E, Mejía ME, Morales M (2015). High prevalence of intermediate Leptospira spp. DNA in febrile humans from urban and rural Ecuador. Emerg Infect Dis..

[12] Schiff SJ, Kiwanuka J, Riggio G, Nguyen L, Mu K, Sproul E, Bazira J, Mwanga-Amumpaire J, Tumusiime D, Nyesigire E (2016). Separating putative pathogens from background contamination with principal orthogonal decomposition: evidence for Leptospira in the Ugandan neonatal septisome. Front Med.

[13] Atherstone C, Picozzi K, Kalema-Zikusoka G (2014). Seroprevalence of Leptospira hardjo in cattle and African buffalos in southwestern Uganda. Am J Trop Med Hyg.

[14] Evangelista KV, Coburn J (2010). Leptospira as an emerging pathogen: a review of its biology, pathogenesis and host immune responses. Fut Microbiol.

[15] Shaked Y, Shpilberg O, Samra D, Samra Y (1993). Leptospirosis in pregnancy and its effect on the fetus: case report and review. Clin Infect Dis.

[16] De Vries SG, Visser BJ, Nagel IM, Goris MGA, Hartskeerl RA, Grobusch MP (2014). Leptospirosis in Sub-Saharan Africa: a systematic review. Int J Infect Dis.

[17] Torgerson PR, Hagan JE, Costa F, Calcagno J, Kane M, Martinez-Silveira MS, Goris MGA, Stein C, Ko AI, Abela-Ridder B (2015). Global burden of leptospirosis: estimated in terms of disability adjusted life years. PLoS Negl Trop Dis.

[18] Allan KJ, Biggs HM, Halliday JEB, Kazwala RR, Maro VP, Cleaveland S, Crump JA (2015). Epidemiology of leptospirosis in Africa: a systematic review of a neglected zoonosis and a paradigm for ‘One Health’in Africa. PLoS Negl Trop Dis.

[19] Dreyfus A, Dyal JW, Pearson R, Kankya C, Kajura C, Alinaitwe L, Kakooza S, Pelican KM, Travis DA, Mahero M (2016). Leptospira seroprevalence and risk factors in health centre patients in Hoima District. Western Uganda PLoS Negl Trop Dis.

[20] Mugalu J, Nakakeeto MK, Kiguli S, Kaddu-Mulindwa DH (2006). Aetiology, risk factors and immediate outcome of bacteriologically confirmed neonatal septicaemia in Mulago hospital, Uganda. Afr Health Sci.

[21] Kiwanuka J, Bazira J, Mwanga J, Tumusiime D, Nyesigire E, Lwanga N, Warf BC, Kapur V, Poss M, Schiff SJ (2013). The microbial spectrum of neonatal sepsis in Uganda: recovery of culturable bacteria in mother-infant pairs. PLoS ONE.

[22] Bourhy P, Bremont S, Zinini F, Giry C, Picardeau M (2011). Comparison of real-time PCR assays for detection of pathogenic Leptospira spp. in blood and identification of variations in target sequences. J Clin Microbiol.

[23] Schoonman L, Swai ES (2009). Risk factors associated with the seroprevalence of leptospirosis, amongst at-risk groups in and around Tanga city, Tanzania. Ann Trop Med Parasitol.

[24] Crump JA, Morrissey AB, Nicholson WL, Massung RF, Stoddard RA, Galloway RL, Ooi EE, Maro VP, Saganda W, Kinabo GD (2013). Etiology of severe non-malaria febrile illness in Northern Tanzania: a prospective cohort study. PLoS Negl Trop Dis.

[25] Biggs HM, Bui DM, Galloway RL, Stoddard RA, Shadomy SV, Morrissey AB, Bartlett JA, Onyango JJ, Maro VP, Kinabo GD (2011). Leptospirosis among hospitalized febrile patients in northern Tanzania. Am J Trop Med Hyg.

[26] van Well GTJ, Daalderop LA, Wolfs T, Kramer BW (2017). Human perinatal immunity in physiological conditions and during infection. Mol Cell Pediatr.

[27] Rosa MI, dos Reis MF, Simon C, Dondossola E, Alexandre MC, Colonetti T, Meller FO (2017). IgM ELISA for leptospirosis diagnosis: a systematic review and meta-analysis. Cien Saude Colet.

[28] Stoddard RA, Gee JE, Wilkins PP, McCaustland K, Hoffmaster AR (2009). Detection of pathogenic Leptospira spp. through TaqMan polymerase chain reaction targeting the LipL32 gene. Diagn Microbiol Infect Dis.

[29] Ahmed A, Grobusch MP, Klatser PR, Hartskeerl RA (2012). Molecular approaches in the detection and characterization of Leptospira. J Bacteriol Parasitol.

